# Reproducibility and Validity of a Short Food Frequency Questionnaire for Dietary Assessment in Children Aged 7–9 Years in Spain

**DOI:** 10.3390/nu11040933

**Published:** 2019-04-25

**Authors:** Jesus Vioque, Manuela Garcia-de-la-Hera, Sandra Gonzalez-Palacios, Laura Torres-Collado, Leyre Notario-Barandiaran, Alejandro Oncina-Canovas, Raquel Soler-Blasco, Manuel Lozano, Andrea Beneito, Eva-Maria Navarrete-Muñoz

**Affiliations:** 1 Unit of Nutritional Epidemiology, Department of Public Health, History of Medicine and Gynecology, University Miguel Hernández, 03550 Alacant, Spain; vioque@umh.es (J.V.); sandra.gonzalezp@umh.es (S.G.-P.); l.torres@umh.es (L.T.-C.); lnotario@umh.es (L.N.-B.); aoncina@umh.es (A.O.-C.); enavarrete@umh.es (E.-M.N.-M.); 2 Alicante Institute for Health and Biomedical Research (ISABIAL-FISABIO Foundation), 03010 Alicante, Spain; 3 CIBER de Epidemiología y Salud Pública (CIBERESP), Instituto de Salud Carlos III, 28009 Madrid, Spain; 4 Epidemiology and Environmental Health Joint Research Unit, FISABIO−Universitat Jaume I−Universitat de València, 46010 Valencia, Spain; Raquel.soler@uv.es (R.S.-B.); Beneito_and@gva.es (A.B.); 5 Foundation for the Promotion of Health and Biomedical Research in the Valencian Region, FISABIO-Public Health, 46035 Valencia, Spain; Lozano_manrel@gva.es; 6 Department of Surgery and Pathology, University Miguel Hernández, 03550 Alacant, Spain

**Keywords:** diet, nutrient intake, food frequency questionnaire, childhood, validity, 24-hour dietary recall, nutritional biomarker, reproducibility, Mediterranean country

## Abstract

The purpose of this study was to examine if the short semi-quantitative food frequency questionnaire (FFQ) is a reliable and valid tool to assess the diet of Spanish children aged 7–9 years. We collected data from 156 children of the birth cohort INMA (Infancia y Medio Ambiente (Environment and Childhood)). Children’s parents or care-givers completed a 46-item FFQ on two occasions over a 9–12-month period about the children’s diet. To explore the reproducibility of the FFQ, the nutrient and food group intake collected from the both FFQs were compared, while validity was examined by contrasting the nutrient values from the FFQs and the average of three 24-hour dietary recalls (24hDRs) taken in this period, and also with the concentration of several vitamins in the blood (carotenoids, vitamin D and α-tocopherol). Pearson and de-attenuated correlation coefficients were calculated. The average correlation coefficients for nutrient intake’s reproducibility was 0.41, ranging from 0.25 (calcium) to 0.65 (β-carotene), and for food group intake was 0.45, ranging from 0.18 (cereals) to 0.68 (sweetened beverages). Correlation coefficients slightly improved when we compared energy-adjusted intakes. The average correlation coefficients for validity against 24hDRs was 0.34 for energy-adjusted intakes, and 0.39 when de-attenuation coefficients were used. The validity coefficients against the blood concentrations of vitamins were 0.38 for β-cryptoxanthin, 0.26 for lycopene, 0,23 for α-carotene and 0.15 for β-carotene, all of them statistically significant (*p* < 0.05). This study suggests that our brief FFQ is a suitable tool for the dietary assessment of a wide range of nutrients and food groups in children 7–9 years, despite the low to moderate reproducibility and validity observed for some nutrients.

## 1. Introduction

Diet in childhood plays vital role on the risk of chronic processes such as obesity, type II diabetes, hypertension, and cardiovascular diseases in adult life [1,2]. Thus, an accurate assessment of the child dietary intake is crucial to monitor nutritional status, its relationship with other lifestyles, and to elucidate the consequences on health and disease later in life.

In assessing a complex exposure like long-term dietary intake in epidemiological studies, food frequency questionnaires (FFQs) have been suggested as the best option because this tool saves time and money compared to other dietary methods [3]. At present, many FFQs have been developed and validated in different populations around the world, mostly in adult populations [4,5,6] and, to a lesser extent, in other specific populations such as pregnant women [7], children or infants [8,9], adolescents [10,11] or elderly people [12].

To date, few validation studies of FFQs have been published for children aged 6–12 years, of which 11 studies were summarized in a systematic review conducted by Orti-Andrelluchi in 2009 [9] and 14 studies were developed in more recent years [13,14,15,16,17,18,19,20,21,22]. Briefly, these studies were conducted in Europe (*n* = 7), South-America (*n* = 6), Australia (*n* = 5), United States (*n* = 3), Canada (*n* = 2), and Asia (*n* = 3); the majority of FFQs were used to assess macro and micro nutrients, and the length of FFQs encompasses a range between 50 and 160 items. The food records and dietary recalls were the preferred dietary assessment methods used as the reference for comparison with FFQs; and average correlation coefficients between nutrient intake in the validation studies was low–moderate (0.3–0.5).

In Spain, there is no validated FFQs to assess dietary intake of school-aged children (6–12 years). Therefore, the purpose of the present study was to examine if the short semi-quantitative FFQ is reliable and valid against three 24-hour dietary recalls (24hDRs) and several vitamins in blood (carotenoids, vitamin D and α-tocopherol) tool to assess the diet in Spanish children aged 7–9 years.

## 2. Materials and Methods 

### 2.1. Study Population

**Figure 1:**
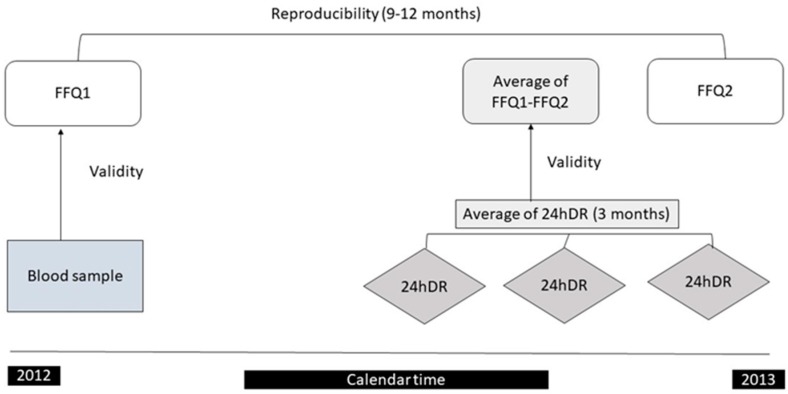
The desing of the validation study among children aged 7 to 9 years of the INMA (Infancia y Medio Ambiente (Environment and Childhood)) project in Valencia, 2012–2013. FFQ: food frequency questionnaire; 24hDR: 24-hour dietary recall.

Participants involved in this validation study were 156 healthy children aged 7–9 years from the Spanish multicenter population-based birth cohort study, the INMA (Infancia y Medio Ambiente (Environment and Childhood)) Project [23]. The present study was conducted in participants from the INMA cohort in Valencia. Briefly, 787 pregnant women delivered a live infant between May 2004 and February 2006, and 461 children attended a planned follow-up visit at the age of 7–9 years. The final sample was estimated to guarantee statistical significance for correlation coefficients of *r* ≥ 0.20 [3]. The design of the validation study is shown in Figure 1. Following standardized procedures, the participants completed with trained nutritionist two FFQs and three 24 hours’ dietary recalls (24hDRs) in a period of 6–12 months and provided a blood sample at baseline. The majority of the children of this study had been also involved in a previous FFQ validation study at 4–5 years old [24]. The research protocol of the study was approved for the ethics committees of the centers involved in the study and the parents of children provided informed consent.

### 2.2. Dietary Assessment: Semi-Quantitative Food Frequency Questionnaire (FFQ)

Child dietary usual intake was retrospectively assessed by trained nutritionists at the 7–9-year follow-up visit using a short semi-quantitative FFQ of 46 food items (available at: http://epinut.edu.umh.es/cfa-46-items/). The FFQ is based on the Harvard questionnaire structure [25] and was derived from previous Spanish FFQs designed to evaluate the dietary intake in pregnancy of the mothers from the INMA-Valencia cohort, and the dietary intake of their children at the age of 4–5 years, showing a good reproducibility and moderate validity [24,26]. The new FFQ was adapted to include food items and portion sizes appropriated for children aged 7 to 9 years and to reduce the number of the items. Parents were asked to report the average frequency of consumption of their children for each item on the FFQ in reference to the past 9–12 months. The questionnaire has nine possible answers, from “never once or less than once a month” to “six or more times a day”. The US Department of Agriculture food composition tables and other Spanish published sources [27,28] were used to estimate nutrient values and total energy intake. In order to obtain average daily nutrient intakes from diet for each child, we multiplied the frequency of consumption of each item by the nutrient content of the portion specified in the FFQ and added the results across all foods. The supplement use was not taken into account to estimate nutrient intake due to a low percentage of children took supplements in our study. We also estimated the mean daily intake of 17 food groups (Table 1).

### 2.3. 24-Hour Dietary Recall (24hDR)

Three non-consecutive 24hDRs, including at least one non-weekday, were collected by trained nutritionists using the United States Department of Agriculture (USDA) Automated Multiple-Pass Method to minimize the forgotten food and beverages consumed, and increase the quality of the dietary information collected [29]. The three 24hDRs were completed during 3-month period by telephone interview; and the third 24hDR was filled out at the same time as the second FFQ. The nutritionist collected the portions, method of preparation and brands to estimate the intake from 24hDRs using the Food Processor Nutrition Analysis Software. The USDA food composition tables and other Spanish published sources [27,28] were used to estimate the nutrient and total energy intake. We used the average of three 24hDRs as a representation of individual intakes.

### 2.4. Nutritional Biomarkers

All the participants provided a non-fasting blood sample at the baseline interview. Vitamin E, D (25-OH-vitamin D), retinol and carotenoids were determined by the General Biochemistry and Hematology Laboratories of the Hospital Puerta de Hierro, Madrid, according to routine quality-controlled standard methods [24,30,31]. In brief, the whole blood samples were centrifuged at 6000 rpm for five minutes to separate blood cells and serum samples within 30 min after of collection. Afterwards, the samples were stored at −80 °C until they were sent to the central laboratory using the standardized protocol by specialized couriers. Serum carotenoids were measured by high performance liquid chromatography (HPLC) with diode array detection using the method which was described by Craft [32], andserum concentrations of α-tocopherol, retinol, vitamin D and individuals’ carotenoids were simultaneously measured by ultra-fast-liquid chromatography [33]. The short and long-term precision and accuracy of the analytical method of laboratory is verified periodically through participation in the Fat-Soluble Quality Assurance Programme conducted by the National Institute of Standards and Technology (NIST; Gaithersburg, MD, USA). Plasma cholesterol was determined to adjust carotenoid concentrations, and the information of serum lutein and zeaxanthin were combined due to this dietary information is grouped in the composition tables.

### 2.5. Statistical Analysis

R software version 3.4.2 (R Foundation for Statistical Computing, Vienna, Austria; http://www.r-project.org) was used to perform statistical analysis. Statistical test were bilateral and significance was determined at level of 0.05. To compare characteristics of the children and their mothers participants (*n* = 156) and non-participants (*n* = 305) in the validation study, we calculated the Chi-square test or Fisher’s exact for categorical variables and Student’s *t*-test for continuous variables. 

We described the nutrient and food groups intakes from FFQ, 24hDRs, and biomarkers using meansand standard deviations. To compare the means between different assessment methods, we used paired Student’s *t*-test.

The nutrient and food group intakes were log transformed to reduce skewness and optimize the normality of the dietary intake distribution. Energy-adjusted intakes were computed using the residual method, where each nutrient is regressed on total calories and the population mean was then added back to the calculated residuals [34]. The plasma carotenoids and tocopherol biomarkers were also adjusted for plasma cholesterol concentrations using the residual method due to these nutrients being transported in plasma lipoproteins. To evaluate the reproducibility of the FFQ, we performed Pearson correlations coefficients between nutrient and food intake measured in two occasions (around 9–12 months). The validity of the FFQ was also computed with Pearson correlation coefficients using as reference method the average of three 24hDRs and biomarkers. To avoid the within-person variation found in the 24hDRs, de-attenuated correlations were calculated using the following formula:

(1) $$\sqrt{1+\{(\frac{S2w}{S2b})/n\}}$$

where *S*2*w* represents within-person variance and *S*2*b* between-person variance for each nutrient and *n* is the number of replicated 24hDRs (in our case *n* = 3) [25]. Due to similar results of Spearman and Pearson correlations, we decided not to present Spearman correlations. We also calculated the percentage of agreement as the proportions of individuals who were classified correctly into the same or adjacent quintile for reproducibility and validity analyses. But, this percentage was not calculated for the food groups’ reproducibility due to the distribution of food group intakes which did not permit us to estimate quintiles.

We examined Bland–Altman plots [35] separately for each of the 31 nutrients to explore the direction of bias across levels of intake and determine the agreement between the two dietary assessment methods used to compare the nutrient intakes (e.g., mean of FFQs vs. average of three 24hDRs).

## 3. Results

The characteristics of the children and mothers of the study population by their participation or non-participation in the validation study are shown in Table 2. Overall, no differences were found between participants or non-participants, except for body mass index (BMI), which was slightly higher in non-participants than in participants (mean 18.0 vs. 17.1; *p* = 0.001). Children participating in the validation study were on average 7.6 years old, of which 45.5% were female and 62.8% were to the school lunchroom once or more times a week. Regarding maternal characteristics of these children, their mothers had a mean age of 30.0 years, more than a third (31.4%) had university studies, 22.4% belonged to a high social class, and 88.5% were born in Spain. In addition, the energy and macronutrients intake at baseline as measured by the first FFQ was very similar between participants and non-participants. The mean daily energy intake in children participants was 1960 Kcals/day and 87 g/day for proteins, 245 g/day for carbohydrates and 75 g/day for total fat.

### 3.1. Reproducibility 

The mean daily nutrient intake, Pearson correlation coefficients and percentage of agreement between the two FFQs completed in approximately a 9–12 month period are presented in Table 3. No differences in nutrients intakes were observed between two FFQs, except for monounsaturated fatty acids, β-cryptoxanthin, vitamin C and vitamin E that showed slightly higher mean consumption in the second FFQ. The average correlation coefficient for crude intakes was 0.41 and for energy-adjusted intakes was 0.46. The correlations for crude intakes ranged from 0.25 for calcium to 0.65 for β-carotene, and all correlations were higher when we used total energy-adjusted intakes. The percentage of agreement of nutrient intake in both FFQs was in average was 69.2%, ranging from 59.6% for monounsaturated fatty acids to 81.4% for β-carotene. The reproducibility of the FFQ for food groups was on average similar to the nutrients; 0.45 for crude intakes and 0.46 in total energy-adjusted intakes (Table 4). We observed a significantly higher mean intake of eggs and fruit and a lower mean intake of dairy in the second FFQ. The lowest reproducibility in crude and total energy-adjusted coefficients were observed for cereal intake (*r* = 0.18 and *r* = 0.15, respectively), and the highest for sweetened beverages (*r* = 0.68 in both coefficients).

### 3.2. Validity

Table 5 displays mean daily nutrient intakes, and Pearson correlations coefficients from the average of two FFQs and the average of three 24hDRs. The mean of nutrient intakes estimated using FFQs were higher than those from the 24hDRs, but the lycopene and vitamin B12 intakes for both methods were very similar between the FFQs and 24hDRs. The average of correlation coefficients for the crude intake, energy-adjusted intake and de-attenuation were 0.31, 0.34 and 0.39, respectively. For crude intakes, the coefficients ranged from r = 0.14 (iodine) to 0.48 (β-cryptoxanthin) whereas the adjustment for energy intake had a similar effect on the levels of correlations. In the comparison of absolute intake assessed by the FFQ and 24hDR, we observed the percentage of agreement was in average of 61.4%, encompassing a range from 51.9% (protein) to 71.2% (retinol).

**Figure 2:**
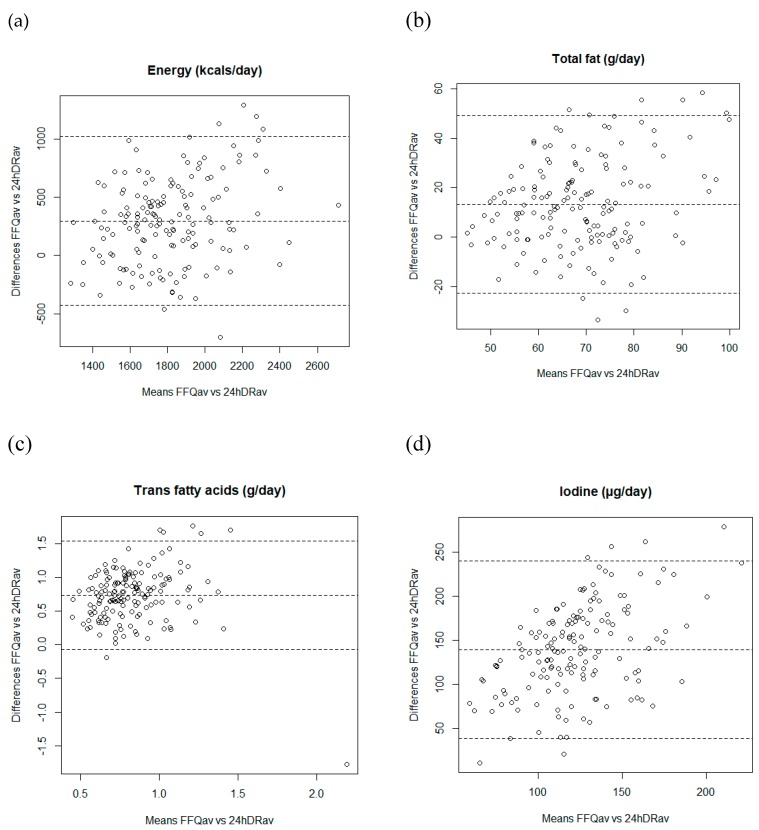
Bland-Altman plots showing the relationship between mean and differences in the daily intake of (**a**) energy; (**b**) total fat; (**c**) trans fatty acids, and (**d**) iodine estimated with the mean of two FFQ and three 24hDRs.

Figure 2 shows the Bland–Altman plots separately for several nutrients (energy, total fat, trans fatty acids, and iodine). When we examined the Bland–Altman plots to explore the validity of the FFQ, the discrepancies between the two dietary assessment methods were equally likely in either direction. The exceptions were trans fatty acids and iodine intake, for which the difference in absolute intakes increased with increasing average intake, with the mean of FFQ producing systematically higher estimates than the average of three 24hDRs.

Table 6 shows the mean daily vitamin intakes and Pearson coefficient correlations of the biochemical validity from the first FFQ and vitamins biomarkers. The average of correlation coefficients for carotenoids was 0.21, ranging from 0.06 (lutein and zeaxanthin) to 0.38 (β-cryptoxanthin). Low correlation coefficients were observed for other vitamins such as vitamin E (*r* = 0.05), retinol (*r* = −0.09) and vitamin D (*r* = 0.06). When we used the total energy-adjusted intake, the correlations increased slightly for most nutrients, except for lutein and zeaxanthin (*r* = 0.04) β-cryptoxanthin (*r* = 0.38) and α-carotene (*r* = 0.23). In addition, when we compared the estimates from FFQ and biomarkers, the average of percentage of agreement of carotenoids was 59.0%. The correlation coefficient between the intake of fruit and vegetables, the main source of carotenoids intake, and carotenoids biomarkers was *r* = 0.19 and the percentage of agreement of both assessment instruments was 60.9%.

## 4. Discussion

This validation study supports the argument that the FFQ may be an acceptable method for dietary assessment in Spanish children aged 7 to 9 years. Our results suggest that the FFQ was moderately reproducible for most nutrients and food group intakes, with correlations ranging from 0.25 to 0.70. The FFQ also showed a reasonable relative validity for most nutrient intakes in comparison to three 24hDRs, and plasma concentration (carotenoids, vitamin E and D), since correlations were in general higher than 0.20 for most of the nutrients. 

Regarding the correlation coefficients to assess the reproducibility of the FFQ for nutrients intakes after 9–12 months’ period, they were consistent with that reported in previous studies conducted in children with similar ages and using shorter periods of time [17,26,36,37]. The results of fruit and vegetable intake reported in our study are similar to those of another study conducted in children living in the Mediterranean area [38]. A previous study conducted with 111 Lebanese children aged 5–10 years reported a higher average of correlation coefficients than observed in our study, 0.62 vs. 0.41 [14], although the better reproducibility could be related to the shorter period of time used between the administration of the two FFQs (one month). Moreover, the number of studies exploring the reproducibility for food groups is very limited [20,37,38]. Field et al. observed coefficients of 0.28 for vegetables and of 0.29 for fruit and vegetables intake [37], considerably lower than those observed in our study (*r* = 0.60 for vegetables, and *r* = 0.67 for fruits). However, Buch-Andersen et al. [39] (*r* = 0.30 to 0.84) and Saeedi et al [20] (*r* = 0.40 to 0.82) reported higher correlations than those observed in our study which may be related to the shorter period of time elapse to complete the two FFQ (2 or 4 weeks), much lower than the 9–12 months’ period used in our study. According to Cade et al. [40] and Willett [25], the range of reproducibility correlation coefficients for nutrient and food groups observed in our study may be considered as adequate, and would suggest that our FFQ is a satisfactorily reproducible tool to assess the nutrient and food intake in Spanish children aged 7–9. 

Dietary record has been proposed as a “gold standard” method to validate other dietary assessment methods such as FFQs [3], although they require a high level of motivation and collaboration from participants. Thus, they may become too burdensome and generate losses to follow-up among other problems (e.g., changes in usual diet). In the present study, we considered it more feasible to use a combination of methods, multiple 24hDRs and biomarkers (for specific nutrients), similar to a previous validation study we performed in children aged 4–5 years from the same project [24]. In accordance with other studies in children with similar age range [13,19], we used three non-consecutive 24hDRs as the reference method to validate our FFQ, which has been considered adequate to estimate energy and nutrient intakes [41]. We did not use a higher number of 24hDRs because this could increase the losses of follow-up and because of the limited resources. In addition, it should be noted that a higher number of recalls can decrease the quality of information collected due to a training effect or the weariness of participants [41]. The mean nutrient intake estimated by 24hDRs observed in our study were around 15%–25% lower than the estimated by the FFQ. Lower estimates for nutrient intakes have been also reported in other studies when comparing 24hDR and FFQ [13,14,16,22,26]. A possible explanation for this lower estimation could be that caregivers did not report the whole children’s food intake, particularly for foods eaten out-of-home, since 62.4% of children in our study reported to use the school lunchroom once a week at least. Despite these disparities in absolute intakes between 24hDR and FFQ, the average correlation coefficients did not change when children attending school lunchroom were excluded from the analysis, which may suggest that the FFQ can be appropriate for ranking participants’ intakes.

The range for correlation coefficients between 24hDR and FFQ observed for crude nutrient estimates in our study (0.14 to 0.48) was similar to those reported by Watson et al. [26] (0.17 to 0.46), Scaglius et al. [22] (−0.04 to 0.59) and Moghames et al. [16] (0.26 to 0.54), and lower than the observed by del Pino et al [13] (0.18 to 0.74). When energy-adjusted nutrient intakes were used, the correlation coefficients tended to increase although not substantially, which may be in part due to the wide variability of the study population characteristics since our study was population-based [34]. Similar findings have been also reported for energy-adjusted correlation coefficients in other studies [16,22,26]. Finally, the use of de-attenuated correlations further increased correlations due to correction of the day to day variation in nutrient intakes

The Bland–Altman diagrams showed no evidence of bias for any of the 31 nutrients examined, except for TFA and iodine, where there was some evidence that the FFQ overestimated intake compared with the 24hDR and that this overestimation increased with increasing average intake. The Bland–Altman diagrams only allow a graphical interpretation and the results should be considered together with other results (e.g., correlation coefficients, cross-classification into quantiles). Thus, our study shows a satisfactory level of agreement in intake ranking between the two dietary methods used.

Several validation studies have reported correlation coefficients between FFQ and serum carotenoids in children to additionally support the biochemical validity (calibration) of the FFQ. In one study carried out in 97 children aged 6–10 years [42], the correlations reported between fruit and vegetables intakes and total serum carotenoids were higher than observed in our study (r = 0.30 vs. r = 0.19), although correlations with specific plasma carotenoids were not performed presented. Our results are comparable to other studies conducted in children [43,44] reporting correlations of plasma concentration for α-carotene (*r* = 0.25 and 0.27 vs. 0.23), β-carotene (0.14 vs. 0.13) and β-cryptoxanthin (0.36 vs. 0.38). In addition, our results for α-carotene, β-carotene and β-cryptoxanthin were similar to other study populations [24,30,31]. However, we found lower correlations between dietary intake and serum lycopene than the reported in other studies in children with similar range of age [43,44]. The low correlation observed in our study between the intake and serum concentration of lutein and zeaxanthin has been also reported in previous studies [24,43], probably suggesting that food sources of these carotenoids were not commonly consumed by children and a much large sample would be required to study this association or that the plasma level for this nutrient is not very sensitive to the intake in children at this age range. Burrows et al. reported strong correlations between dietary and serum carotenoids after adjusting for BMI; however, the correlations did not materially change when we adjusted for BMI [43]. In our study, we also observed very low correlations between dietary and serum for retinol, vitamin D and E which may be due to other non-dietary factors affecting circulation concentrations, efficiency of absorption and metabolism. In fact, retinol concentration is highly regulated by liver stores over a wide range of dietary intakes that can affect serum levels. Also, it has been suggested that plasma concentrations of α-tocopherol may not be a good marker of vitamin E for usually intake in children, and 25OHD may be a poor biomarker of dietary vitamin D since the absorption can be influenced for the sunlight exposure [45,46]. 

A major strength of the present study may be the use of two reference methods (three 24hDRs and biomarkers) with different error sources to validate the FFQ. Another strength was the use of well-trained nutritionists to collect dietary information during the study period and standard protocols for collecting blood samples. Moreover, the study population was a subsample of the children with very similar characteristics to the whole participant children in the INMA study, a population-based birth cohort and, therefore, the results could be more generalizable to children of the same age range. On the other hand, a potential limitation would be that the dietary information collected by FFQs was self-reported by caregivers and they may be not fully aware of all the food items eaten by their children, especially foods eaten out-of-home. However, we observed that correlations did not change when we excluded the children who had school meals. In addition, the majority of the caregivers in this study had already participated in a prior validation study of a similar FFQ for dietary assessment at the age of 4–5 years and, therefore, they could provide more precise information in this new study. Children were present at the moment of interview and were able to provide additional details about the menu of school lunchroom and other foods and beverages eaten out-of-home. In order to minimize the possible influence of the nutritionists in the dietary report of caregivers, they were instructed in particular to collect the dietary information of the first and second FFQs avoiding subjective opinions. In addition, the 24hDRs were completed by telephone following the USDA Automated Multiple-Pass Method to minimize the forgotten food and beverages consumed by the children in the previous day.

## 5. Conclusions

In conclusion, this study shows that this short FFQ covering a wide range of foods and nutrients may be a useful instrument to evaluate dietary intake for most nutrients and food groups in Spanish children aged 7 to 9 years. The questionnaire showed acceptable reproducibility and validity, comparable to other validation studies with children of similar age range.

## Figures and Tables

**Table 1:** Description of the food items included in the food groups.

Food Groups (Number of Foods Items)	Foods
Dairy Products (4)	whole dairy products; semi-skimmed, skimmed and fortified dairy products; Petit Suisse; cheese.
Eggs (1)	Eggs
White meat (1)	chicken or turkey
Red meat (1)	beef, pork or lamb
Processed meat (3)	sausages; ham, salami and others; serrano ham
Fatty fish (3)	swordfish, bonito, and fresh tuna; small oily fish (mackerel, sardine; anchovy); canned sardine or mackerel
Lean fish (1)	A assorted or mixed fried fish (hake; gilthead sea bream and sole)
Seafood (2)	clams, mussels, squid, octopus, shellfish (cramps, shrimps, lobster); surimi and other fish-based food products
Fruits (2)	oranges; other fruit (apples; banana; pears; watermelon; melon; peach; kiwi; cherries, strawberries)
Vegetables (2)	raw vegetables (tomatoes; onions; lettuces; peppers and carrots) and cooked vegetables (spinach; cabbage, cauliflower or broccoli; carrots or squash; eggplant, zucchini, or cucumber; green, red, or yellow peppers)
Nuts (1)	almonds, walnuts, peanuts and other types of nuts
Legumes (1)	lentils, chickpeas, beans, peas, and green beans
Cereals and Pasta (2)	breakfast cereals; corn, rice and pasta
Bread (2)	white and whole breads
Potatoes (2)	frozen French fry; homemade boiled/stew
Sweets and sugar (5)	biscuits and baked goods; biscuits and baked goods with chocolate; peanut butter (e.g., Nutella/Nocilla); chocolate/cocoa powder; candies, marmalade, and honey
Sweetened beverages (3)	packages juices; sugar soft drinks and artificially soft drinks

**Table 2:** Characteristics of children and their mothers who attended a 7–9 year follow up distinguishing between participants or no participants in the validation study (Yes/No) of the INMA-Valencia cohort study.

	Participation in the Validation Study		*p*-Value ^1^
	Yes (*n* = 156)	No (*n* = 305)	
**Maternal Characteristics**			
Age (years), mean (standard deviation (SD))	30.0 (4.0)	30.8 (4.2)	0.058
Education (University studies), %	31.4	29.5	0.645
Social class (I + II; high), %	22.4	19.7	0.469
Country of birth (Spain), %	88.5	86.2	0.560
**Child Characteristics**			
Age (years), mean (SD)	7.6 (0.1)	7.6 (0.2)	0.666
Sex (female), %	45.5	51.8	0.237
Body Mass Index (Kg/m^2^), mean (SD)	17.1 (2.7)	18.0 (3.0)	0.001
School lunchroom (≥1 time/week), %	62.8	64.8	0.682
Energy (Kcals/day), mean (SD)	1960 (459)	1902 (435)	0.188
Proteins (g/day), mean (SD)	87 (18)	85 (18)	0.391
Carbohydrates (g/day), mean (SD)	245 (65)	233 (62)	0.054
Fats (g/day), mean (SD)	75 (20)	74 (19)	0.725

^1^*p*-values from Student’s *t*-test (continuous variables) and from Chi-square or Exact Fisher tests (categorical variables).

**Table 3:** Mean daily nutrient intakes and Pearson correlation coefficients in two FFQs in children aged 7–9 years of the INMA-Valencia study (*n* = 156).

Nutrient Intakes (units/day)	FFQ1 ^1^	FFQ2 ^1^	*p*-Value ^2^	Pearson Coefficient Correlations between FFQ1 and FFQ2		% of Agreement ^5^
	Mean (SD)	Mean (SD)		r ^3^	r adj.^4^	
Energy (kcals/day)	1953 (458)	1962 (410)	0.852	0.34		65.4
Protein (g/day)	87 (18)	85 (17)	0.344	0.29	0.48	62.8
Total carbohydrates (g/day)	245 (65)	243 (60)	0.698	0.36	0.40	68.6
Dietary fiber (g/day)	20.3 (6.1)	21.1 (6.1)	0.226	0.43	0.51	66.7
Cholesterol	286 (69)	284 (69)	0.849	0.43	0.47	71.8
Total fat (g/day)	73 (20)	77 (20)	0.125	0.35	0.32	67.3
SFA (g/ day)	25.7 (7.9)	25.6 (7.6)	0.903	0.34	0.42	62.2
MUFA (g/day)	28.2 (8.7)	31.2 (9)	0.004	0.33	0.24	59.6
PUFA (g/day)	12.6 (3.7)	13.4 (4)	0.101	0.42	0.49	67.9
Omega 3 (g/day)	1.3 (0.4)	1.4 (0.4)	0.411	0.43	0.57	67.9
Omega 6 (g/day)	11.1 (3.3)	11.8 (3.7)	0.083	0.42	0.47	67.9
Trans fatty acid (g/day)	1.2 (0.4)	1.2 (0.4)	0.386	0.32	0.42	75.6
Retinol (μg/day)	452 (216)	440 (174)	0.594	0.44	0.47	75.6
α-carotene (μg/day)	816 (647)	908 (717)	0.236	0.64	0.63	80.8
β-carotene (μg/day)	2721 (2038)	3083(2259)	0.138	0.65	0.65	81.4
β-Cryptoxanthin (μg/day)	159 (102)	203 (145)	0.002	0.54	0.49	71.8
Lutein + Zeaxanthin (μg/day)	1451 (780)	1481 (939)	0.758	0.54	0.55	73.7
Lycopene (μg/day)	2800 (1355)	2942 (1682)	0.412	0.44	0.45	70.5
Vitamin B6 (mg/day)	1.7 (0.7)	1.7 (0.6)	0.962	0.47	0.49	71.2
Folate (μg/day)	213 (50)	222 (61)	0.164	0.41	0.39	70.5
Vitamin B12 (μg/day)	6.4 (2.3)	6.3 (1.9)	0.497	0.39	0.42	69.2
Vitamin C (mg/day)	78 (41)	95 (58)	0.002	0.51	0.46	72.4
Vitamin D (μg/day)	4.4 (1.6)	4.4 (1.5)	0.789	0.47	0.54	69.9
Vitamin E (mg/day)	7.6 (2.3)	8.4 (2.6)	0.002	0.39	0.44	63.5
Calcium (mg/day)	1135 (344)	1105 (310)	0.429	0.25	0.35	65.4
Iron (mg/day)	13 (5.2)	13 (4.5)	0.942	0.45	0.46	72.4
Magnesium (mg/day)	284 (64)	282 (64)	0.752	0.31	0.43	64.7
Potassium (mg/day)	2730 (607)	2731 (645)	0.989	0.33	0.41	69.2
Sodium (mg/day)	2812 (703)	2746 (552)	0.355	0.30	0.43	64.7
Zinc (mg/day)	10.1 (2.6)	9.8 (2.2)	0.326	0.32	0.40	66.0
Iodine (μg/day)	196 (57)	186 (55)	0.112	0.48	0.59	73.1
Average of correlation coefficients				0.41	0.46	69.2

^1^ FFQ1 & FFQ2, the same FFQ was firstly administered at baseline (FFQ1) and secondly (FFQ2), between 9 to 12 months later; ^2^ *p*-value from paired *t*-tests; ^3^ r: coefficient correlations after nutrient crude intakes were log-transformed; ^4^ r adj: correlation coefficient using nutrient intakes adjusted for total energy; ^5^ Percentage of children classified in the same or an adjacent quintile in nutrient crude intakes; SFA, saturated fatty acids; MUFA, monounsaturated fatty acids; PUFA, polyunsaturated fatty acids; all correlation coefficients were statistically significant, *p* < 0.01.

**Table 4:** Mean daily food group intakes and Pearson correlation coefficients in two FFQs among children aged 7–9 years of the INMA-Valencia study (*n* = 156).

Food Groups (g/day)	FFQ1 ^1^	FFQ2 ^1^	*p*-Value ^2^	Pearson Coefficient Correlations between FFQ1 and FFQ2	
	Mean (SD)	Mean (SD)		r ^3^	r adj. ^4^
Dairy products	561 (244)	505 (211)	0.031	0.25	0.32
Eggs	22 (9)	24 (9)	0.046	0.59	0.57
White meat	40 (15)	38 (14)	0.497	0.42	0.42
Red meat	27 (15)	25 (15)	0.282	0.40	0.40
Processed meat	35 (22)	32 (14)	0.138	0.37	0.39
White fish	24 (13)	23 (13)	0.560	0.58	0.57
Blue fish	16 (14)	18 (14)	0.288	0.61	0.63
Seafood	9 (15)	7 (9)	0.131	0.45	0.45
Vegetables	95 (73)	103 (82)	0.392	0.60	0.60
Fruit	160 (107)	218 (145)	<0.001	0.67	0.65
Nuts	4 (4)	4 (4)	0.785	0.28	0.30
Pulse	33 (21)	31 (19)	0.458	0.57	0.56
Cereals	82 (50)	81 (38)	0.846	0.18	0.15
Potatoes	40 (20)	39 (19)	0.628	0.34	0.32
Bread	98 (51)	99 (48)	0.872	0.35	0.39
Sweet and sugar	75 (52)	68 (47)	0.200	0.34	0.35
Sweetened beverages	141 (153)	137 (142)	0.807	0.68	0.68
Average of correlation coefficients				0.45	0.46

^1^ FFQ1 and FFQ2, the same FFQ was firstly administered at baseline (FFQ1) and secondly (FFQ2), between 9 to 12 months later. ^2^ *p*-value from paired *t*-tests; ^3^ r: coefficient correlations after food groups intakes were log-transformed; ^4^ r adj: correlation coefficient using food groups intakes adjusted for total energy; the correlations coefficients had a *p*-value < 0.01 when *r* ≥ 0.20, and a *p* value < 0.05 when 0.15 ≤ *r* ≤ 0.19.

**Table 5:** Mean daily nutrient intakes and Pearson correlation coefficients from the average of two FFQs and three 24hDRs among children aged 7–9 years of the INMA-Valencia study (*n* = 156).

Nutrients (units/day)	FFQav ^1^	24hDRav ^2^	*p*-Value ^3^	Pearson Coefficient Correlations between FFQav and 24hDRav			% of Agreement ^7^
	Mean (SD)	Mean (SD)		r ^4^	r adj. ^5^	r de-att. ^6^	
Energy (kcals/day)	1957 (354)	1662 (271)	<0.001	0.31			62.2
Protein (g/day)	86 (14)	70 (13)	<0.001	0.25	0.36	0.42	51.9
Total carbohydrates (g/day)	244 (52)	211 (43)	<0.001	0.40	0.34	0.39	65.4
Dietary fiber (g/day)	20.7 (5.2)	12.9 (3.6)	<0.001	0.32	0.29	0.30	57.1
Cholesterol (g/day)	285 (58)	229 (71)	<0.001	0.23	0.28	0.31	58.3
Total fat (g/ day)	75 (16)	62 (13)	<0.001	0.24	0.32	0.34	57.7
SFA (g/ day)	25.6 (6.3)	21.9 (5.2)	<0.001	0.35	0.32	0.35	60.3
MUFA (g/day)	29.7 (7.2)	24.1 (5.8)	<0.001	0.18	0.26	0.28	58.3
PUFA (g/ day)	13 (3.2)	9.8 (3.1)	<0.001	0.24	0.38	0.50	58.3
Omega 3 (g/day)	1.3 (0.4)	1 (0.3)	<0.001	0.24	0.31	0.37	57.7
Omega 6 (g/day)	11.5 (2.9)	8.7 (2.8)	<0.001	0.25	0.38	0.54	63.5
Trans fatty acids (g/day)	1.2 (0.3)	0.5 (0.3)	<0.001	0.44	0.48	0.60	67.3
Retinol (μg/day)	446 (165)	374 (205)	0.001	0.46	0.45	0.57	71.2
α-Carotene (μg/day)	862 (601)	200 (301)	<0.001	0.32	0.31	0.33	61.5
β-Carotene (μg/day)	2902 (1899)	955 (944)	<0.001	0.43	0.43	0.45	64.1
β- Cryptoxanthin (μg/day)	181 (102)	94 (95)	<0.001	0.48	0.48	0.54	64.7
Lutein + Zeaxanthin (μg/day)	1466 (726)	655 (924)	<0.001	0.24	0.24	0.26	57.1
Lycopene (μg/day)	2871 (1300)	2679 (2028)	0.319	0.22	0.20	0.20	60.9
Vitamin B6 (mg/day)	1.7 (0.5)	1.4 (0.5)	<0.001	0.46	0.49	0.50	68.6
Folate (μg/day)	218 (47)	151 (50)	<0.001	0.25	0.28	0.29	60.3
Vitamin B12 (μg/day)	6.4 (1.8)	5.8 (5.7)	0.223	0.28	0.30	0.39	64.1
Vitamin C (mg /day)	87 (41)	53 (35)	<0.001	0.43	0.44	0.56	64.1
Vitamin D (μg/day)	4.4 (1.3)	2.7 (1.8)	<0.001	0.22	0.24	0.28	59.6
Vitamin E (mg/day)	8 (2)	5.1 (1.7)	<0.001	0.19	0.23	0.26	57.7
Calcium (mg/day)	1120 (264)	898 (240)	<0.001	0.38	0.45	0.50	59.6
Iron (mg/day)	13 (4.1)	10.7 (4.3)	<0.001	0.41	0.37	0.43	66.7
Magnesium (mg/day)	283 (52)	211 (39)	<0.001	0.36	0.39	0.41	61.5
Potassium (mg/day)	2731 (509)	2124 (462)	<0.001	0.32	0.48	0.51	63.5
Sodium (mg/day)	2779 (504)	2210 (511)	<0.001	0.25	0.29	0.32	57.7
Zinc (mg/day)	9.9 (2)	8.1 (1.8)	<0.001	0.39	0.29	0.31	62.8
Iodine (μg/day)	191 (48)	80 (44)	<0.001	0.14	0.19	0.20	57.8
Average of correlation coefficients				0.31	0.34	0.39	61.4

^1^ FFQav: Average of FFQ1 and FFQ2; ^2^ 24hDRav: average of the three 24hDRs; ^3^ *p*-value from paired *t*-tests; ^4^ r: coefficient correlations after nutrient intakes were log-transformed; ^5^ r adj: correlation coefficient using energy-adjusted nutrient intakes; ^6^ r de-att: de-attenuated correlation coefficients after nutrient intakes were log-transformed and energy-adjusted; ^7^ percentage of children classified into the same or an adjacent quintile; the correlations coefficients had a *p*-value < 0.01 when *r* ≥ 0.20, and a *p*-value < 0.05 when 0.15 ≤ *r* ≤ 0.19.

**Table 6:** Mean daily vitamin intakes Pearson correlation coefficients from the first FFQ and vitamin biomarkers in children aged 7–9 years of the INMA-Valencia study (*n* = 156).

Nutrients	FFQ1 ^1^	Vitamin Biomarkers	Pearson Coefficient Correlations between FFQ1 and Plasma Concentrations		Agreement (%) ^4^
	Mean (SD)	Mean (SD)	r ^2^	r adj ^3^	
Vitamin E	7.6 (2.3)	961 (173)	0.05	0.10	56.4
Vitamin D	4.4 (1.6)	66 (20)	0.06	0.11	53.2
Retinol	452 (216)	25 (6)	−0.09	0.05	51.9
Carotenoids					
Lutein + Zeaxanthin	1451 (780)	13.6 (6.3)	0.06	0.04	50.0
β- Cryptoxanthin	159 (102)	14 (12)	0.38	0.38	65.4
Lycopene	2800 (1355)	28 (11)	0.24	0.26	62.8
α-Carotene	816 (647)	7.6 (4.7)	0.23	0.23	55.1
β-Carotene	2721 (2038)	24 (16)	0.13	0.15	56.4
Average of correlation coefficients			0.21	0.21	59.0
Fruits and vegetables vs. total carotenoids	140 (153)	88 (34)	0.19	0.19	60.9

^1^ FFQ was firstly administered at baseline (FFQ1); ^2^ r: coefficient correlations after nutrient intakes were log-transformed and cholesterol adjusted for carotenoids and vitamin E; ^3^ r adj: correlation coefficient using energy-adjusted nutrient intakes and cholesterol adjusted for carotenoids and vitamin E; ^4^ percentage of children classified into the same or an adjacent quintile the correlations coefficients had a *p*-value < 0.01 when *r* ≥ 0.20, a *p*-value < 0.05 when 0.15 ≤ *r* ≤ 0.19, and a *p*-value > 0.05 when *r* < 0.15.
